# An Experimental Study of Step Test Index Combined With Heart Rate Variability in Estimating Maximum Oxygen Uptake in Women With Drug Use Disorder

**DOI:** 10.3389/fphys.2020.00322

**Published:** 2020-04-30

**Authors:** Kun Wang, Tingran Zhang, Yiyi Ouyang, Haonan Jiang, Meichen Qu, Li Peng, Jiong Luo

**Affiliations:** ^1^Research Centre for Exercise Detoxification, College of Physical Education, Southwest University, Chongqing, China; ^2^Key Laboratory of Physical Fitness Evaluation and Motor Function Monitoring, College of Physical Education, Southwest University, Chongqing, China

**Keywords:** women, methamphetamine users, step test, cardiopulmonary exercise test, VO_2max_, heart rate variability

## Abstract

**Background:**

Maximal oxygen uptake (VO_2max_), a vital physiological indicator, has been widely used in many fields. In recent years, the measurement method of VO_2max_ has been widely explored in various populations, but few studies have been conducted for women drug abusers. For the importance of VO_2max_ in the formulation of aerobic exercise intensity for drug users, the present study estimated VO_2max_ using the step test index combined with heart rate variability in women with drug use disorder.

**Methods:**

Forty women methamphetamine (MA) users without cardiovascular disease and dyskinesia participated in a cardiopulmonary exercise test (CPX) and a 3-minute step test. Each of them performed a heart rate variability (HRV) monitoring test after the step test, and VO_2max_ was estimated by step test index and HRV.

**Results:**

(1) The step test index had a significant positive correlation with VO_2max_. The standard deviation of normal-to-normal interval (SDNN) had a significant positive correlation with VO_2max_ and a significant positive correlation with the step test index; (2) the R-square values of the estimated VO_2max_ by step test index and post-SDNN for overall MA users were 0.29 and 0.22, with an accuracy of 93.19 and 92.85%, respectively; (3) the R-square values of the estimated VO_2max_ by step test index and post-SDNN in group I were 0.27 and 0.36, respectively, with an accuracy of 94.04 and 93.99%. The R-square value of the estimated VO_2max_ by step test index in group II was 0.44, with an accuracy of 92.65%, however, post-SDNN cannot adequately estimate the VO_2max_ in group II; and (4) there was no significant difference in VO_2max_ obtained by CPX, step test index, or post-SDNN, regardless of overall or grouping variable analysis.

**Conclusion:**

The 3-minute step test combined with HRV can estimate the VO_2max_ of women MA users to a certain extent, but the size and the coverage of the sample size should be further considered. In the future, more methods, such as machine learning or artificial neural networks, should be used.

## Introduction

In recent years, the drug abuse problem has become increasingly devastating on a global scale. According to relevant data, in 2016 alone, about 275 million individuals used drugs at least once worldwide, which accounted for 5.6% of the global population aged 15–64 years (UNODC, 2018). By the end of 2018, there were 2.504 million drug users in China, accounting for 0.18% of the country’s total population (National Anti-Drug Committee Office, 2019). Among them, the use of methamphetamine (MA)-based synthetic drugs has become the most popular form of drug abuse. As well known, the long-term use of MA can cause functional damage to the nervous system and the cardiovascular system, then produce strong drug dependence (Panenka et al., 2013; Gong et al., 2019), and contribute to arrhythmia, tachycardia, and myocardial ischemia. Those symptoms eventually lead to heart damage (Varner et al., 2002; Li et al., 2009), and MA smoking not only severely impairs the physical and the mental health of individuals but also poses a significant threat to public safety (Liu et al., 2010; Mcketin et al., 2013). Therefore, research on MA users has been a research hotspot in various fields. Among them, cardiovascular complications, such as hypertension, acute coronary syndrome, aortic dissection, and pulmonary hypertension, are associated with MA abuse. Besides that, an acute high-dose MA administration can cause significant toxic damage to the heart, kidney, and liver of rats, accompanied by increased levels of oxidative stress (OS) (Li et al., 2016; Tan, 2019). It can be seen that the research on the cardiopulmonary function of MA users is significant.

Maximal oxygen uptake (VO_2max_) is currently well recognized as an important factor in testing the overall function of the cardiovascular system and cardiorespiratory fitness. It is not only a risk indicator for estimating cardiovascular deterioration and monitoring the functional status of patients (Aspenes et al., 2011) but also an important indicator for assessing the body’s aerobic capacity (Bosquet et al., 2002).

The American Heart Association ranked cardiopulmonary fitness as the fifth vital sign after heart rate, respiration, blood pressure, and body temperature in 2016. Subsequently, cardiorespiratory fitness was widely evaluated, and the importance of VO_2max_ measurement and evaluation has drawn more attention (Coquart et al., 2016). The traditional way to measure VO_2max_ is to use the cardiopulmonary exercise test (CPX) combined with a treadmill, bicycle ergometer, or step test to directly monitor the breathing gas of the subject during exhaustive exercise and then evaluate it according to established criteria. The data measured by this method is highly reliable and regarded as the “gold standard” for measuring VO_2max_. However, this method has many limitations, such as being time-consuming, non-portable, and expensive equipment. Besides that, it is not suitable for special populations such as the elderly, weak, sick, and young (Liu and Dong, 2013; Dong et al., 2017), so researchers have been looking for alternatives. Currently, in a healthy population, the more common test methods include the 12-minute run (e.g., Cooper’s 12-minute run test, CRT) (Hu et al., 2019), 1,000-m run (Li et al., 2010), 6-minute walk (Jones et al., 2017), step test (de Andrade et al., 2012b; Li et al., 2015), and 20-m shuttle run (Paradisis et al., 2014). In studies on specific populations, the CRT is often used to estimate cardiopulmonary function in asthmatic and obese children (Calders et al., 2008; Weisgerber et al., 2009), but there have been other studies that have questioned CRT. Then, the Astrand–Rhyming method is used to estimate breast cancer and the cardiopulmonary function of schizophrenia (Vancampfort et al., 2014; Mijwel et al., 2016). These were all validated, but few studies have been conducted on cardiopulmonary function indicators such as VO_2max_ in populations with drug use disorder.

Among the many test methods mentioned above, based on the advantage of the step test in terms of the low requirements on equipment and test settings, it can be used for extensive sample testing, and it has become a commonly used indirect measurement method of VO_2max_ (Dong et al., 2017; Fan et al., 2019). However, in previous studies, due to the difference in height and exercise intensity of the step and the differences in the physiology and muscle strength of the subjects themselves, in a Chinese national physical fitness monitoring, whether the step test accurately reflected cardiopulmonary endurance was questioned (Wang and Deng, 2003). Therefore, some studies have shown that, in step test, the physiological analysis should be back to itself, that is, take the cardiovascular indicators as an objective basis (de Andrade et al., 2012a). It is well known that the cardiovascular system is mainly regulated by the endocrine and the autonomic nervous system (ANS), and the ANS can control blood distribution by heart rate, stroke volume, and vascular resistance. Should the ANS be out of adjustment, and not timely monitored and diagnosed, those conditions are likely to cause adverse effects such as heart failure (Kishi, 2012). HRV reflects the degree of variation in each heartbeat interval, the nervous tension, and the balance regulation of the sympathetic nervous system and the parasympathetic nervous system. As a non-invasive indicator for evaluating the ANS functional status, HRV has been widely used in physiology and clinical medicine in recent years (Qu et al., 2006).

In summary, the importance of VO_2max_ is widely recognized, so the way of testing and application become diversified, but it has not been fully verified in a specific population such as MA users. Moreover, owing to the relatively simple evaluation method, the reliability and the validity of the results are questioned as well (Peng, 2011). Besides that, the number of years of using drugs is one of the critical indicators for assessing the physical and the mental health of people with MA dependence. The longer the years of using the drug, the stronger the addiction on drugs tend to be (Chen et al., 2017) and the more influential the body’s tolerance to medication is (Jiang, 2006)–the cognitive level will be significantly reduced. That of women will be considerably lower than that of males (Zhang et al., 2016). Based on the discussion above, we believe that the 3-minute step test combined with HRV can be used to estimate and evaluate the VO_2max_ of women MA users, thus providing a theoretical basis and an actual reference for the development of exercise rehabilitation prescription for MA users.

## Materials and Methods

### Participants

Forty-five women MA users were recruited from the Education and Correction Centre for Drug Abusers in Chongqing, China. They were selected to participate in this trial according to the following inclusion criteria: (1) age: 18–40 years, (2) level of education ≥primary school, (3) under the Education and Correction Center and more than 3 months of mandatory abstention and rehabilitation, (4) drug users following the DSM-IV criteria by structured interview judgment, (5) no history of loss of consciousness due to head injury, (6) no psychosis or immediate family members suffering from mental illness history, (7) no heart or cardiovascular disease, or (8) no physical disability or contraindication of moderate-intensity (or above) aerobic exercise.

To further study the effect of drug addiction experience on VO_2max_, according to the years of drug use experience, 40 subjects were divided into two groups: group I (drug use experience <10 years, *n* = 26) and group II (drug use experience ≥10 years, *n* = 14). All the subjects signed an informed consent after understanding the content and requirements of this study and then completed the corresponding questionnaire (Table 1). This study was conducted according to the guidelines laid down in the Declaration of Helsinki and was approved by the ethics committee of the Southwest University Hospital (201905). The written informed consent form was obtained from every participant before enrolling them in the study.

**TABLE 1:** Basic data statistics of the participants [M ± SD / *n* (%), *N* = 40]

**Variables**		**Group I**	**Group II**
**Demographic information**			
Gender	Women	26 (65%)	14 (35%)
Age (year)		26.35 ± 4.45	31.50 ± 4.11
Body height (m)		1.55 ± 0.05	1.54 ± 0.05
Body weight (kg)		57.19 ± 6.92	58.49 ± 7.43
BMI		23.70 ± 2.50	24.39 ± 2.41
Marital status	Married	2 (5%)	3 (7.5%)
	Unmarried	15 (37.5%)	3 (7.5%)
	Divorced	9 (22.5%)	8 (20%)
Educational degree	Primary school	2 (5%)	3 (7.5%)
	Middle school	19 (47.5%)	6 (15%)
	High school	4 (10%)	5 (12.5%)
	College or above	1 (2.5%)	0
Occupation	Unemployed	11 (27.5%)	9 (22.5%)
	Self-employed	2 (5%)	0
	Civil servant	0	1 (2.5%)
	Office clerks	8 (20%)	2 (5%)
	Others	5 (12.5%)	2 (5%)
Monthly income	>1,000 yuan/person	14 (35%)	5 (12.5%)
**Drug user relevant information**			
Methamphetamine use experience (year)		4.96 ± 1.54	12.86 ± 4.33
Methamphetamine use frequency (time/week)		3.00 ± 0.89	3.64 ± 0.84
Cigarette smoking	Yes	17 (42.5%)	13 (32.5%)
	Experience (year)	6.19 ± 1.98	11.36 ± 2.98
Alcohol drinking	Yes	13 (32.5%)	10 (25%)
	Experience (year)	6.27 ± 1.71	9.86 ± 2.28
Times of compulsory abstinence (times)		1.38 ± 0.64	1.86 ± 0.86

*Group I, methamphetamine users with an experience <10 years; Group II, methamphetamine users with an experience ≥10 years; BMI, body mass index [BMI is calculated by dividing the body weight in kilograms by the height in meters squared (kg/m^2^)].*

### Measurement Instruments

#### Cycle Ergometers

The stationary cycle ergometers (Lode-906900, Dutch) were used for pedaling, and the pedaling rhythm during the movement was maintained at 60–70 rpm. If the pedaling rhythm lasts for 3 s below 60 rpm, the pedal should be stopped immediately. The exercise loading program was no-load exercise (0 w) during the first 2 min, and then the exercise load was increased linearly for each minute until the subject reached the exhausted state.

#### Step Test

The step test was performed using a Kedao step tester (TZCS-3, China). The subject first needed to step on a single-step step with a height of 30 cm, and the frequency of going up and down step was kept at 30 times/min, and the time was 3 min. Immediately after completion, the subject should sit in front of the tester and then insert the middle finger into the clamp. In the pulse clamp, the signal light flashes synchronously with the subject’s heartbeat, and the step test index was automatically generated after 3.5 min.

#### Portable Gas Exchanger

A portable gas exchanger (PKSP-11, China) equipped with a mask and a gas analyzer to collect gas was used in this study. Before each test, the instrument was calibrated with standard gas and an individual calibration cylinder. The gas exchanger can record the gas exchange status during the entire exercise test procedure.

#### Heart Rate Variability Monitoring System

The HRV monitoring system (Healink Ltd., Bengbu, China) was used in this study, which can effectively monitor the time domain and the frequency domain indicators of the sympathetic and the parasympathetic nerves of the subjects. The bandwidth of the device was 0.5–40 Hz, and the sampling rate was 400 Hz. V5-lead was employed, and the measuring electrodes were Ag/AgCl disposable electrocardiogram (ECG) electrode pads (Junkang Ltd., Shanghai, China). The relevant time domain and frequency domain HRV indices were recorded. Mainly, the time domain indices include the standard deviation of normal-to-normal intervals (SDNN) and the root mean square successive difference (RMSSD) (Wierig et al., 2018). Among them, the SDNN was the most commonly used parameter (Wang et al., 2019), which was calculated by the mean of all normal R–R periods and its standard deviation. During the test, the subject should sit quietly, with a non-invasive two-lead electrocardiogram attached, for a test duration of 5 min.

### Experimental Procedure

**FIGURE 1:**
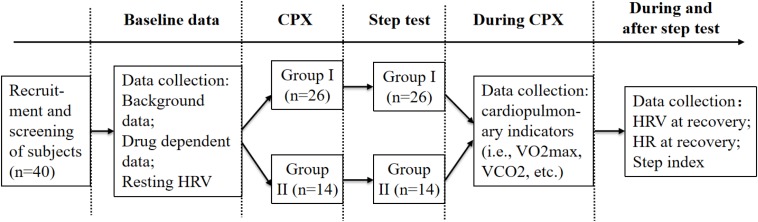
Flow chart of the experimental procedure.

The 40 MA users screened for the trial were required to complete the following three tests separately: baseline test, cardiopulmonary exercise test, and 3-minute step test. For the accuracy of the test data, all subjects were divided into groups I and II according to the years of drug use experience after completing the baseline test and then followed by CPX and the 3-minute step test (Figure 1).

#### The Baseline Test

(1) background information such as demographic data, drug abuse history, and drug-dependent survey; and (2) heart rate and HRV at resting. The subjects should avoid staying up late and performing vigorous physical activity 1 day before the test of HRV. The testing was done for 5 min.

#### CPX

After wearing the gas exchanger, the subject sat on the stationary bicycle ergometer, the height of which was adjusted for it to be suitable for the specific testing of individuals and was thus prepared for the test. During the pedal process, the test system automatically and simultaneously recorded the gas metabolism parameters, such as VO_2_, of each breath of the subject immediately and continuous verbal encouragement was provided to the subject until the termination conditions of the test according to the American College of Sports Medicine (ACSM) were reached. Appropriate safety precautions were adopted throughout the testing; the subject’s response was closely monitored and the test was stopped immediately if there was any discomfort.

#### The 3-Minute Step Test

The subjects should also avoid staying up late and performing a vigorous physical activity 2 days before the test. Before the formal experiment, the subjects sat for 3 min in a quiet and gentle environment with their eyes closed and while remaining silent. When officially getting started, the subject went up and down the step for 3 min at a fixed speed for 30 times/min (each going up and down as a time), and the accompaniment music was used as the auditory feedback. On completion of the 3-minute step test, the subject immediately sits on a chair and takes a rest, while the step test index is recorded. OMRON T10 wrist blood pressure monitor was used to record the heart rate on three occasions and the blood pressure during the recovery period as the subject keeps relaxing and adjusting the breathing with the eyes closed and while keeping silent. The HRV was recorded after 5 min of rest, and the recording time was 5 min. After the data acquisition was completed, the step test ended. If the stepping rhythm was three consecutive beats slower, the step test was stopped immediately, and the HRV of the stepping time and the recovery period were recorded.

#### Termination Test Condition

This study was based on the recommendations of the ACSM. When the subjects experienced the following indications in the baseline test and the step test, the test was stopped: (1) subject’s desire to stop, (2) fatigue, shortness of breath, leg cramps, wheezing, dizziness, nausea, and so forth, (3) abnormal heart rate, such as heart rate was too fast or too slow, and (4) VO_2_ reaches the platform (VO_2_ increasing rate is less than 150 ml/min).

### Data Analysis and Processing

In this study, the ECG viewer software (HeaLink Ltd., Bengbu, China) used for RRI extraction of HRV data to pre-process the ECG data, statistical analysis, and automatic correction of ECG data was Heartlink software (HeaLink Ltd., Bengbu, China). Firstly, the RR interval sequence was uniformly resampled by a detrend algorithm. Then, the uniformly sampled sequence was subjected to layer-by-layer wavelet decomposition to filter out the extremely low-frequency components, thereby achieving the purpose of removing non-stationary trends. Secondly, by setting a threshold value (sliding window width was *w* = 50), the points above the threshold value in the calculation result of the impulse rejection filter were replaced by the average value of the surrounding points to filter out abnormal pulses. The data were processed and analyzed by SPSS 21.0 for Windows. The test of normality of all variables was carried out using the Shapiro–Wilk test, and all test variables followed the normal or appropriate normal distribution criteria, so the parametric tests have been used. The descriptive statistics of continuous variables have been shown in mean ± standard deviation (M ± SD). The CPX and HRV indicators of different drug users were tested by the independent-sample *t*-tests, Pearson correlation analysis on VO_2max_, SDNN, and step test index. A general linear regression analysis of VO_2max_ has been established by the step test index and SDNN, then paired-sample *t*-test and multivariate analysis of variance [groups (group I, group II) × 3 methods (CPX, step test index, and SDNN)] were used to detect the difference in VO_2max_ obtained in different ways. Bonferroni correction was used in pairwise comparisons. The significance levels of all statistical tests were set to *α* = 0.05.

## Results

### Descriptive Statistics of Cardiopulmonary Function and Heart Rate Variability Indicators at Resting

Table 2 shows that, from the perspective of groups, the pre-SDNN, pre-RMSSD, pre-LF, pre-HF, pre-LF/HF, and HR had no significant difference in the quiet state between groups (*P* > 0.05). Similarly, there were no significant differences in VO_2max_, VCO_2_, AT, and VE in CPX measurements between groups (*P* > 0.05).

**TABLE 2:** Difference of CPX and resting HRV indicators between groups (M ± SD, *N* = 40).

**Variables**	**Group I (*n* = 26)**	**Group II (*n* = 14)**	***T***	***P***
**CPX**				
VO_2_ max (ml/min/kg)	26.232.67	25.503.01	0.79	0.44
VCO_2_ (ml/min/kg)	1,653.69239.46	1,627.29236.69	0.33	0.74
AT (L/min)	15.084.03	16.074.03	−0.91	0.37
VE (L/min)	54.818.42	56.039.86	−0.41	0.68
**Pre-HRV**				
Pre-SDNN (ms)	55.1515.46	48.2611.80	1.45	0.16
Pre-RMSSD (ms)	53.8516.54	53.1020.02	1.23	0.90
Pre-LF (ms^2^)	62.6212.10	63.0710.50	−0.12	0.91
Pre-HF (ms^2^)	39.5411.15	38.4310.64	0.31	0.76
Pre-LF/HF	1.710.61	1.780.61	−0.37	0.71
Resting HR (beats/min)	72.049.47	70.216.66	0.64	0.53

*CPX, cardiopulmonary exercise test; Pre-HRV, heart rate variability indicators at resting before the exercise; VO_2max_, maximum oxygen uptake; VCO_2_, carbon dioxide ventilation; AT, anaerobic threshold; VE, ventilatory rate; SDNN, standard deviation of normal-to-normal interval; RMSSD, root mean square of the standard deviation; LF, low frequency (refers to 0.04–0.15 Hz); HF, high frequency (refers to 0.15–0.40 Hz); LF/HF, the ratio of low frequency to high frequency; HR, heart rate.*

### Descriptive Statistics of Heart Rate and Heart Rate Variability Indicators at Recovery After the 3-Minute Step Test

After the 3-minute step test, the HRV test was carried out at a recovery of 5–10 min. The results in Table 3 show that there was a significant difference (*T* = 2.08, *P* < 0.05) in post-SDNN between group I (52.78 ± 11.65 ms) and group II (45.16 ± 9.81 ms); no significant difference was found in post-RMSSD, post-LF, post-HF, and LF/HF between groups (*P* > 0.05). There were no significant differences in the three HR tests (*P* > 0.05) during the recovery. Similarly, no significant difference was observed in the step indexes calculated by the formula (*P* > 0.05).

**TABLE 3:** Difference in Heart rate and HRV indicators during recovery after step test by group (M ± SD, *N* = 40).

**Variables**	**Group I (*n* = 26)**	**Group II (*n* = 14)**	***T***	***P***
**Post-HRV**				
Post-SDNN (ms)	52.7811.65	45.169.81	2.08	0.04*
Post-RMSSD (ms)	41.0218.65	43.0318.86	−0.32	0.75
Post-LF (ms^2^)	56.6213.63	55.4314.04	0.26	0.80
Post-HF (ms^2^)	48.6914.85	43.7914.37	1.01	0.32
Post-LF/HF	1.220.35	1.350.34	−1.05	0.30
**HR (recovery)**				
HR(1)	108.3512.90	111.5016.55	1.45	0.16
HR(2)	103.3812.94	98.5015.30	1.07	0.29
HR(3)	98.1212.63	93.2113.48	1.14	0.26
Step index	58.497.51	56.534.46	0.89	0.38

*Post denotes 5–10 min at recovery after the step test; recovery denotes the recovery period after the step test; HR(1) denotes the heart rate of 1–1.5 min at recovery; HR(2) denotes the heart rate of 2–2.5 min at recovery; HR(3) denotes the heart rate of 3–3.5 min at recovery; ^∗^ denotes *P* < 0.05.*

### Test of the Overall Correlation on Estimated VO_2max_ by the 3-Minute Step Test Combined With Heart Rate Variability

The correlation analysis showed (Table 4) that (1) VO_2max_ was significantly positively correlated with pre-SDNN (*r* = 0.36, *P* < 0.05) and VO_2max_ was significantly positively correlated with post-SDNN (*r* = 0.47, *P* < 0.01); VO_2max_ was significantly positively correlated with the step test index (*r* = 0.54, *P* < 0.001); (2) pre-SDNN was significantly positively correlated with post-SDNN (*r* = 0.78, *P* < 0.001), and pre-SDNN and step test index showed a significant positive correlation (*r* = 0.50, *P* < 0.01); and (3) post-SDNN and step test index showed a significant positive correlation (*r* = 0.39, *P* < 0.01). There was a significant positive correlation between variables, which provided a theoretical basis for the subsequent regression analysis.

**TABLE 4:** Analysis table of overall correlation test (*N* = 40).

	***M***	**SD**	**1**	**2**	**3**	**4**
VO_2_ max	25.98	2.78	–			
Pre-SDNN	52.74	14.51	0.36*	–		
Post-SDNN	51.11	11.52	0.47**	0.78***	–	
Step index	57.80	6.61	0.54***	0.50**	0.39**	–

* *P* < 0.05, ** *P* < 0.01, *** *P* < 0.001.

### Linear Regression Analyses on Estimated VO_2max_ by the 3-Minute Step Test Combined With Heart Rate Variability

#### Overall Linear Regression Analysis on Estimated VO_2max_

**FIGURE 2:**
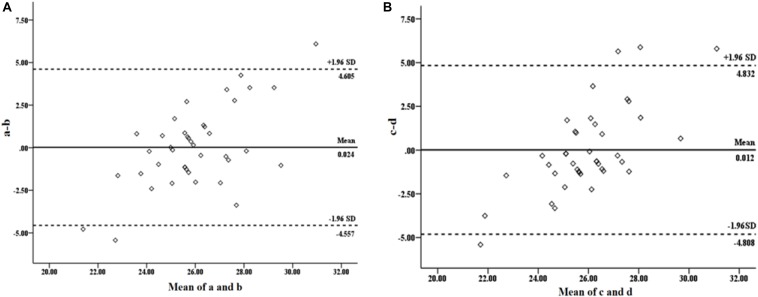
Bland–Altman test plot of the estimated and the measured VO_2max_. **(A)** The measured VO_2max_ by cardiopulmonary exercise test (CPX) and estimated VO_2max_ value by the step test. **(B)** The measured VO_2max_ by CPX and the estimated VO_2max_ by HRV.

The results of this study showed that the Bland–Altman plot drawn based on the step index (Figure 2A) and SDNN (Figure 2B) has some singular values, causing some errors to exceed the acceptable range, which may be due to the small sample size. Research showed that when the sample size was small, the sampling error will be relatively large, that the larger the sample size, the wider the data cover, and that the smaller the LoA CI range, the easier it was to reach a better consensus conclusion. Similarly, Hopkins (2004) pointed out that the Bland–Altman plot will show a proportional deviation under the simulation data without systematic proportional deviation. Therefore, in order to ensure the goodness-of-fit of the regression equation, we combined the Bland–Altman method and linear regression analysis to estimate the VO_2max_ of women MA users and removed singular values in the subsequent analysis.

**FIGURE 3:**
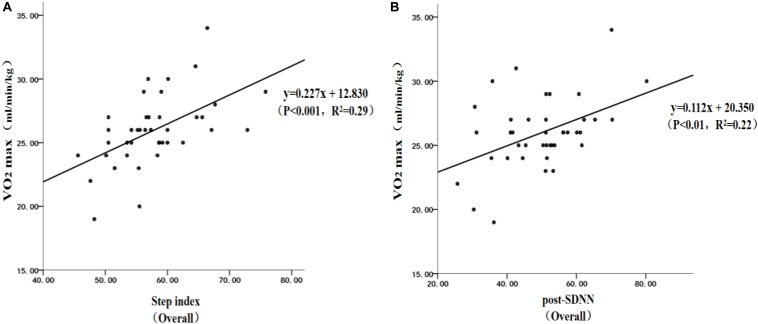
Linear regression analysis on the estimated VO_2max_ by the step test combined with hear rate variability (HRV): **(A)** estimated VO_2max_ by the step test; **(B)** estimated VO_2max_ by HRV.

Linear regression analysis found that, for the subjects as a whole, the 3-minute step test combined with HRV can estimate VO_2max_ positively regardless of the years of drug use experience (Figure 3). The individual performance was as follows: (1) the step test index had a significant positive estimated effect on VO_2max_ (Figure 3A). The regression formula was *y* = 0.227*x* + 12.830 (*P* < 0.001, *R*^2^ = 0.29); and (2) post-SDNN had a significant positive estimated effect on VO_2max_ (Figure 3B). The regression formula was *y* = 0.112*x* + 20.350 (*P* < 0.01, *R*^2^ = 0.22). Both equations above can significantly estimate VO_2max_, which indicated that it was reasonable to estimate the VO_2max_ of drug users by a 3-minute step test combined with HRV.

#### Linear Regression Analysis on VO_2max_ of Drug Users by Years of Drug Use

Based on the fact that the number of years of drug use is often regarded as one of the critical variables in previous studies, to explore further the optimal estimating formula for VO_2max_ of drug users, 40 subjects have been divided into two groups by drug use years, and then linear regression analysis was performed on the two groups using step test combined with HRV.

**FIGURE 4:**
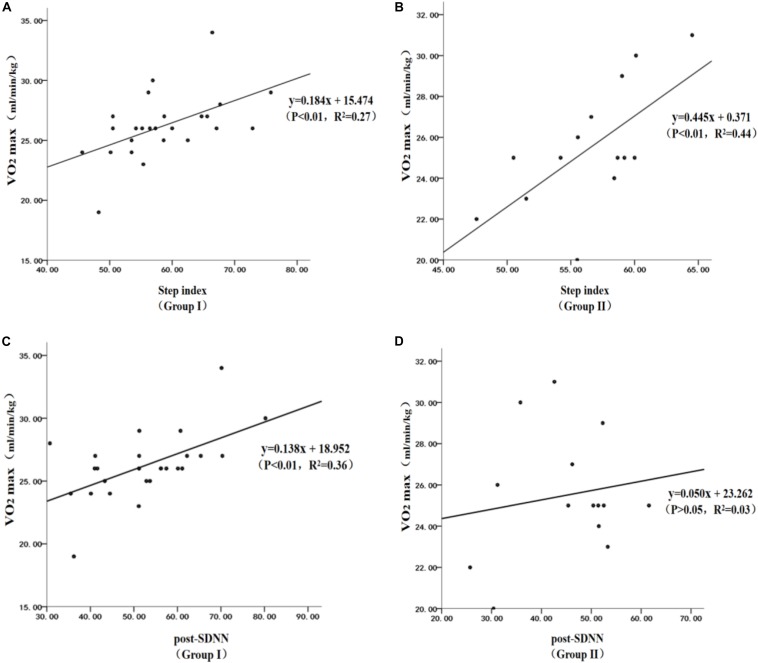
Scatterplot of the linear regression of the estimated VO_2max_ in methamphetamine (MA) users: **(A)** estimated VO_2max_ in MA users by the step index in group I, **(B)** estimated VO_2max_ in MA users by the step index in group II, **(C)** estimated VO_2max_ in MA users by SDNN in group I, and **(D)** estimated VO_2max_ in MA users by SDNN in group II.

Linear regression analysis found that the 3-minute step test combined with HRV can effectively estimate VO_2max_ in groups I and II drug users (Figure 4). The specific performances were as follows: (1) the step test index had significant positive estimated effect on VO_2max_ in group I (Figure 4A), and the regression formula was *y* = 0.184*x* + 15.474 (*P* < 0.01, *R*^2^ = 0.27). The step test index had a significant positive estimated effect on VO_2max_ in group II (Figure 4B), and the regression formula was *y* = 0.445*x* + 0.371 (*P* < 0.01, *R*^2^ = 0.44); (3) post-SDNN had a significant positive estimated effect on VO_2max_ in group I (Figure 4C), and the regression formula was *y* = 0.138*x* + 18.952 (*P* < 0.01, *R*^2^ = 0.36); and (4) it was worth noting that post-SDNN did not have a significant estimated effect on VO_2max_ in group II (*P* > 0.05, Figure 4D), and the regression formula was *y* = 0.050*x* + 23.262 (*P* > 0.05, *R*^2^ = 0.03). It was indicated that the 3-minute step test combined with HRV had a certain difference in the estimation of VO_2max_ on users by years of using drugs.

### Reliability and Validity Test on the Value of VO_2max_ Estimated by the 3-Minute Step Test Combined With Heart Rate Variability and Cardiopulmonary Exercise Test Direct Measurement

#### Difference Test Between Cardiopulmonary Exercise Test Measured Value and VO_2max_ Estimated Value

**FIGURE 5:**
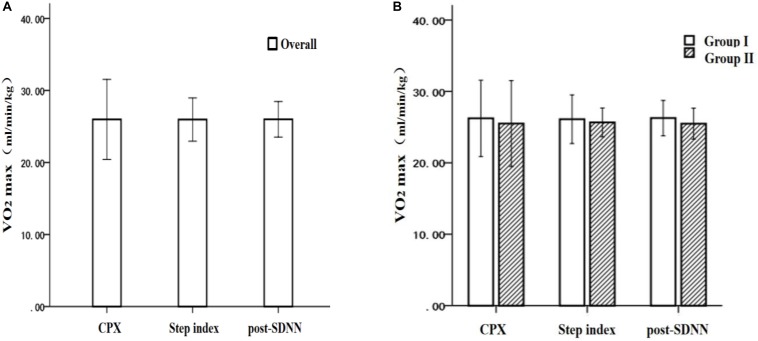
The difference in VO_2max_ by the cardiopulmonary exercise test, the step test index, and the post-SDNN: **(A)** overall comparison and **(B)** grouping comparison.

Figure 5 shows that (1) the paired-sample *T*-test was used to, respectively, analyze the overall VO_2max_ difference of the CPX measured value, the step test index measured value, and post-SDNN estimated value (Figure 5A). It was found that there was no significant difference between the estimated value of the step test index (25.95 ± 1.50 ml/min/kg) and the measured value of CPX (25.98 ± 2.78 ml/min/kg) (*T* = 0.07, *P* > 0.05), the difference between the estimated value of post-SDNN (25.96 ± 1.29 ml/min/kg) and the measured value of CPX (25.98 ± 2.78 ml/min/kg) (*T* = 0.03, *P* > 0.05) was not significant, and the difference between the estimated value of the step test index (25.95 ± 1.50 ml/min/kg) and the estimated value of post-SDNN (25.96 ± 1.29 ml/min/kg) (*T* = −0.05, *P* > 0.05) was also not significant; and (2) A 2 × 3 multivariate analysis of variance was used to analyze the differences in CPX measurements, step test index, and post-SDNN estimated value in the different groups of subjects (groups I and II) (Figure 5B). The results showed that there was no significant difference between the CPX measured values (*F* = 0.62, *P* > 0.05) and the step test index estimated values (*F* = 0.80, *P* > 0.05) in the two groups. In contrast, the group differences of the post-SDNN estimated value (*F* = 4.33, *P* < 0.05) was significant, but because post-SDNN cannot effectively estimate the VO_2max_ in group II, the difference in the results of the reorganization was thus excluded. The further simple effect analysis found that, in group I, there was no significant difference between the estimated step test index (26.11 ± 1.70 ml/min/kg) and the CPX measurement (26.23 ± 2.67 ml/min/kg) (*T* = 0.28, *P* > 0.05), the difference between the estimated value of post-SDNN (26.26 ± 1.30 ml/min/kg) and the measured value of CPX (26.23 ± 2.67 ml/min/kg) (*T* = −0.07, *P* > 0.05) was not significant, and the difference between the estimated value of the step test index (26.11 ± 1.70 ml/min/kg) and the estimated value of post-SDNN (26.26 ± 1.30 ml/min/kg) (*T* = −0.47, *P* > 0.05) was also not significant. In group II, there was no significant difference between the estimated step test index (25.66 ± 1.01 ml/min/kg) and the measured CPX value (25.50 ± 3.01 ml/min/kg) (*T* = −0.25, *P* > 0.05), the difference between the estimated value by post-SDNN (25.41 ± 1.10 ml/min/kg) and the measured value of CPX (25.50 ± 3.01 ml/min/kg) (*T* = 0.11, *P* > 0.05) was not significant, and the difference between the estimated value of the step test index (25.66 ± 1.01 ml/min/kg) and the estimated value of post-SDNN (25.41 ± 1.10 ml/min/kg) (*T* = 0.74, *P* > 0.05) was also not significant.

#### Precision Analysis on Estimated VO_2max_ Value by Step Test Combined With Heart Rate Variability

Table 5 shows that in terms of the reliability and the validity tests of the estimated value of VO_2max_ by the 3-minute step test combined with HRV, the accuracy of the estimate was more than 90% regardless of the overall or grouping estimation. Among the whole groups I and II, the regression equation with the step test index as the independent variable had better accuracy of an estimate than that with post-SDNN as the independent variable. Still there was no significant difference between the two methods. The specific performance was as follows: (1) overall: the R-square value of step test index was 0.29, and the accuracy of the estimated value was 93.19%; the R-square value of post-SDNN was 0.22, and the accuracy of the estimated value was 92.85%; (2) group I: the R-square value of step test index was 0.27, and the accuracy of the estimated value was 94.04%; the R-square value of post-SDNN was 0.36, and the accuracy of the estimated value was 93.99%; and (3) group II: the R-square value of step test index was 0.44, and the accuracy of the estimated value was 92.65%, but the post-SDNN cannot adequately estimate VO_2max_ (*P* > 0.05).

**TABLE 5:** The accuracy of the estimated VO_2max_ by the step test index and the post-SDNN (*N* = 40).

	***Stepindex***				***Post*−*SDNN***			
	***Regressionequation***	**R-square**	**95%*CI***	**Accuracy**	***Regressionequation***	**R-square**	**95%*CI***	**Accuracy**
Overall	*y* = 0.227*x* + 12.830	0.29	(0.12, 0.37)	93.19%	*y* = 0.112*x* + 20.350	0.22	(0.02, 0.18)	92.85%
*GroupI*	*y* = 0.184*x* + 15.474	0.27	(0.06, 0.34)	94.04%	*y* = 0.138*x* + 18.952	0.36	(0.05, 0.24)	93.99%
*GroupII*	*y* = 0.445*x* + 0.371	0.44	(0.15, 0.77)	92.65%	*y* = 0.050*x* + 23.262	0.03	(−0.22, 0.21)	–

*The accuracy of the estimated value was calculated by 1 − [(cardiopulmonary exercise test measured value − estimated value) / cardiopulmonary exercise test measured value)] × 100%.*

## Discussion

This study was a pilot study to estimate the VO_2max_ of women MA users by the 3-minute step test combined with HRV and confirmed that it had great potential for the application of the estimated VO_2max_ value by 3-minute step test. So far, it was a more effective and timely way to monitor the physiological status of drug users and to promote the development of scientific drug use disorder rehabilitation.

### VO_2max_ Closely Related to Heart Rate Variability Indicators in Methamphetamine Users

This study found that the VO_2max_ and the HRV of MA users were significantly correlated before and after exercise, and it indicated that SDNN was significantly positively correlated with VO_2max_ at rest and at. It was well known that VO_2max_ was one of the critical indicators to evaluate cardiopulmonary function. It can adequately reflect cardiopulmonary endurance and the cardiovascular system status and is a critical monitoring factor for preventing cardiovascular diseases. However, MA use can seriously damage the cardiovascular system. High-dose MA can cause arrhythmia, myocardial ischemia, and acute hypertension and even lead to myocardial infarction (Karch et al., 1999; Swalwell and Davis, 1999). Fortunately, as one of the non-invasive ECG monitoring indicators, the HRV has drawn much attention in recent years (de Andrade et al., 2012b) as it can be used not only as a diagnostic indicator for cardiovascular disease but also as a useful estimating indicator for complications, such as arrhythmia and sudden cardiac death, in reducing myocardial infarction (Malik et al., 1989). Among them, SDNN is the most commonly used indicator in HRV, which belongs to the time domain analysis. It can calculate the average value of all normal R–R periods and obtain the standard deviation. It can be seen that the combination of VO_2_ max and HRV indicators for detecting the cardiovascular system and the cardiopulmonary function for special populations such as MA users may have significant application value.

### Analysis of Estimated VO_2max_ by 3-Minute Step Test Combined With Heart Rate Variability

#### Novelty Analysis

Previous studies have shown that the correlation coefficient between the VO_2max_ and the Astrand nomogram combined with PWC_170_ two-stair step test was low, resulting in low cardiopulmonary endurance validity (Qiu et al., 2001; Bland et al., 2012), and the estimated VO_2max_ value by the 6-minute two-stair step test was smaller than by CPX (Fan et al., 2019). The classic Harvard step test has a relatively wide range of application, and the test reliability is much higher than that of the Ohio University Step Test and Queens College step test. However, the Harvard step test intensity is relatively high, and relying merely on the step test index cannot adequately reflect the age-related change in cardiac function and the gender differences (Wang and Deng, 2003). For example, in a 3-minute step test for the elderly, using stepping frequency of 20 times/min and a step height of 20 and 25 cm, respectively, the results showed that there was significant correlation between the step test index of 25 cm and VO_2max_. Still the step test index of 20 cm has no significant correlation with VO_2max_ (Li et al., 2015). The cause was that, with the change of the step height, the rhythm and the duration of the motion, and even the calculation of the step test index, the effect of load on the subjects varies as well (Buckley et al., 2004). The importance of establishing a specific step test model for different populations can be seen. Therefore, this study was geared to improve the applicability and the accuracy of the step test in women drug users. On the one hand, based on the classic Harvard step test, the step height was adjusted to 30 cm, the duration of 3 min was still used, and VO_2max_ was estimated by step test combined with HRV, which was relatively novel. The exercise intensity of this test was calculated according to the formula developed by ACSM, which was consistent with the fundamental physiological characteristics of women MA users. On the other hand, based on the grouping of the critical variable of years of drug use experience, this study further explored whether the estimated VO_2max_ of women drug users by the 3-minute step test combined with HRV had a difference in drug use years and hoped to find a scientific method to effectively estimate the VO_2max_ of women drug users.

#### Analysis of Effectiveness

The results of this study showed that there was a significant positive correlation between step index by 3-minute step test combined with HRV, SDNN, and VO_2max_ as measured by CPX. This finding was consistent with numerous studies (Santo and Golding, 2003; Pogliaghi et al., 2014; Guo et al., 2016; Liu et al., 2017). Regardless of the overall or the grouping estimate, the valid regression equation can be established according to the step test index or SDNN as the independent variable, and there was no significant difference between the estimated value and the CPX measurement value. The regression equation established by them had a higher accuracy on the estimated VO_2max_, value. Among them, the accuracy of the estimated value by the regression equation developed by the step test index in group I was slightly higher than that in group II. The accuracy of the estimate of the regression equation established by the SDNN in group I was somewhat higher than in the whole, but there was no significant difference. It was indicated that the estimated VO_2max_ of MA users by the 3-minute step test combined with HRV was reasonable and practical, and there was no difference in drug use years. Previous studies have shown that the step test had excellent measurement reliability and can effectively evaluate the cardiovascular system function, and the step test index was more sensitive to the motion effect than VO_2max_ (Wang and Deng, 2003). However, relying merely on the step test index to determine VO_2max_ was gradually being questioned. In the process of the experiment, it should be returned to the physiological symptoms themselves to better estimate aerobic fitness such as VO_2max_ and to exclude some other disturbing factors. Therefore, the reliability and the validity can be improved (Ryhming, 1953). Thus, the estimating method of combining the HRV monitoring with the 3-minute step test can not only effectively monitor the HRV of the MA users after the step test but also improve the reliability and the validity of the step test to estimate VO_2max_. Compared with the traditional cycle ergometer test, the up and down step exercise can mobilize more muscles, enhance blood oxygen circulation, and thus increase oxygen consumption (Fan et al., 2019). It can be seen that the 3-minute step test combined with HRV was reasonable and useful for estimating VO_2max_.

#### Limitation Analysis

The subjects of this study were restricted to women MA users, so the finding was only applicable to the women MA user population, which had certain limitations. During the test, the step test results were more dependent on the subject’s initiative, and the exercise intensity was not easy to control, which will affect the test results to some extent (Liu et al., 2003). At the same time, although the 3-minute step test was more stable during the test, there were individual differences in the height and the weight of the subject, and the subject’s pedaling rhythm was different, which inevitably caused the subject to move up and down the center of gravity in an effort of overcoming the subject’s weight (Yuan et al., 2012), however, previous studies have indicated that there was no significant correlation between the step test index and the height, the weight, and the length of the lower limbs in children and adolescents (Meyers, 1969). In terms of HRV, there was a lack of more effective indicators to evaluate VO_2max_ during the step test. It is still difficult to avoid the difference between the test indicators by using the heart rate and the step test index combined with SDNN.

## Conclusion and Prospects

### Conclusion

The 3-minute step test index combined with HRV can adequately estimate the VO_2max_ of MA users. It indicated that both the step test index and SDNN could adequately estimate the VO_2max_ of women MA users, and the accuracy of the estimates was 93.19 and 92.85%, respectively. For the women using MA for less than 10 years, the accuracy of the estimated value of VO_2max_ by the step test index and SDNN was 94.04 and 93.99%, respectively, while for 10 years and above, only the step test index had a significant estimating power (the accuracy of estimate was 92.65%). There was no significant difference in the estimated VO_2max_ by CPX, 3-minute step test, or SDNN. It indicated that the results obtained by the three methods were in good agreement.

### Prospects

(1) This study used a cross-sectional study, so it cannot obtain the long-term effect of the 3-minute step test on cardiopulmonary function indicators, such as VO_2max_ in women MA users, and cannot monitor the autonomic nervous system through HRV for a long time. In the future, longitudinal studies should be performed to reveal their inherent traits and connections.

(2) In future research, male MA users can be recruited to carry out comparative studies by gender, and further scientific research can be conducted to determine whether VO_2max_ has gender differences.

## Data Availability Statement

All datasets generated for this study are included in the article/supplementary material.

## Ethics Statement

The studies involving human participants were reviewed and approved by Southwest University Hospital. The patients/participants provided their written informed consent to participate in this study.

## Author Contributions

All authors designed this study, contributed and approved the final manuscript. KW, TZ, and JL carried out the experiment procedure. MQ recruited the individuals with drug addicts. YO and HJ undertook the statistical analysis and graphical representation of the data. LP and JL revised the draft.

## Conflict of Interest

The authors declare that the research was conducted in the absence of any commercial or financial relationships that could be construed as a potential conflict of interest.
